# Quality of life following cochlear implants in elderly recipients: a prospective cohort study

**DOI:** 10.1017/S0022215124001695

**Published:** 2025-04

**Authors:** Haisam Shah, Yuning Xue, Hamna Rehman, Glen Watson, Kerry Hitos, Melville da Cruz

**Affiliations:** 1 Sydney Medical School, The University of Sydney, Westmead, NSW, Australia; 2 Department of Otolaryngology, Head and Neck Surgery, Royal Hallamshire Hospital, Sheffield, UK; 3 Westmead Hospital, The University of Sydney, NSW, Australia; 4 Westmead Research Centre for Evaluation of Surgical Outcomes, Westmead Hospital, Sydney, NSW, Australia; 5 Research and Education Network, Westmead Hospital, New South Wales, Australia; 6 Department of Otolaryngology, Westmead Hospital, NSW, Australia

**Keywords:** cochlear implants, deafness, hearing loss, quality of life

## Abstract

**Background:**

Deafness is a leading cause of disability worldwide. This prospective cohort study investigates the impact of cochlear implants on self-reported quality of life in post-lingually deaf adults.

**Methods:**

The self-administered 36-item World Health Organization Disability Assessment Schedule 2.0 and the Speech, Spatial, and Qualities of Hearing Scale questionnaires were prospectively used to investigate the impact of cochlear implants in 98 post-lingually deaf adults aged more than or equal to 50 years.

**Results:**

Quality of life improved post-cochlear implant in the cumulative scores and scores for all domains of the Speech, Spatial, and Qualities of Hearing Scale (*p* < 0.05). QoL improved post-cochlear implant in the sub-domains related to cognition and participation in society of the World Health Organization Disability Assessment Schedule 2.0 (*p* < 0.05), but there was no significant difference in the cumulative score. Subgroup analysis showed improvement in the participation in society domain only and only in males and participants aged younger than 75 years (*p* < 0.05).

**Conclusion:**

Cochlear implant improves quality of life in post-lingually deaf adults.

## Introduction

The World Health Organization reports hearing loss as the third leading cause of disability worldwide, affecting 35 to 45 per cent of adults over 50 years old.^1^ Hearing loss can lead to anxiety, depression and social isolation. It negatively impacts communication skills, reduces quality of life (QoL) and has been linked to cognitive decline and the development of dementia, particularly in older patients.^2^ It is crucial to study hearing loss interventions to assess their impact on QoL and hearing loss-associated sequelae.

Cochlear implants (CIs) remain the gold standard intervention for patients with moderate to profound sensorineural hearing loss, who do not benefit from hearing aids. CIs are cost-effective and efficacious in all age groups; however, the multifaceted implications of CI on QoL remain underappreciated.^3^^,^^4^ QoL is based on the complex interplay between sensory, demographic (e.g., duration of deafness) and cognitive factors. Traditional objective audiometric and speech recognition measurements are unable to capture the holistic benefits of CIs on QoL, which prompted the advent of self-reported QoL questionnaires.

Many self-reported questionnaires have been established to investigate the impact of hearing loss interventions, like CI, on QoL. Disease-specific questionnaires such as the Nijmegen Cochlear Implant Questionnaire (NCIQ), Hearing Handicap Inventory for the Elderly (HHIE) and the Speech, Spatial, and Other Qualities of Hearing (SSQ) are more sensitive for hearing-specific QoL, but do not consider other domains of QoL indirectly affected by sensory function.^5^^–^^7^ Generic questionnaires such as the Short Form 36 (SF-36), World Health Organization Quality of Life Scale (WHOQOL), Health Utilities Index Mark II (HUI2) and Mark III (HUI3) and the World Health Organization Disability Assessment Schedule 2.0 (WHODAS 2.0) are independent of disease aetiology, allowing assessment across a broader range of QoL domains and therefore enabling comparisons between different diseases, cohorts and clinical contexts.^8^^–^^10^

Studies using self-reported questionnaires in several developed countries have shown that CIs improve QoL.^11^ This knowledge has been crucial in guiding the management of hearing-impaired older adults in Australia. However, these studies cannot fully represent the experiences of Australian patients due to differences in culture, population characteristics and health care systems. This study aimed to investigate the impact of CIs on QoL in Australians aged more than or equal to 50 years with post-lingual sensorineural hearing loss, the specific QoL domains that were affected and the impact of gender and age.

## Materials and methods

### Inclusion and exclusion criteria

The pre-defined inclusion criteria required participants to be aged more than or equal to 50 years, literate in English, have moderate to profound post-lingual sensorineural hearing loss and be eligible for CI surgery through a standard selection process at the Sydney Cochlear Implant Center (now NextSense). Non–English-speaking participants were excluded as the QoL questionnaires were in English and limited understanding of the language may introduce random errors into the study. No specific inclusion criteria related to gender and ethnicity were set, and no efforts were made to specifically include or exclude Aboriginal and Torres Strait Islander participants.

### Study design

This is a prospective, non-randomized cohort study. Power calculations were performed based on the assumption that CI would improve QoL by at least 30 per cent, which indicated that a sample size of at least 60 participants was needed.

**Figure 1.**
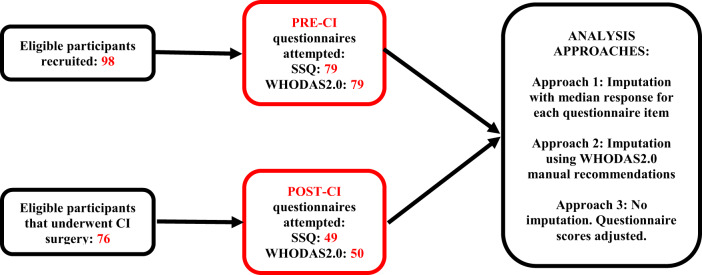
Flow chart depicting study design, the number of participants that attempted each survey, and the three different analytic approaches used for handling missing data.

We invited 98 eligible patients from Westmead Public and Private Hospitals in Sydney, Australia, to participate in the study (Figure 1). Participants were mailed the SSQ and 36-item WHODAS 2.0 questionnaires to complete before CI surgery. Follow-up SSQ and WHODAS 2.0 questionnaires were mailed to participants 12 months post-operation. Participants who did not have CI surgery were still included in the study, as their pre-CI QoL scores were used for data analysis. Participants self-administered all questionnaires without assistance from study personnel. Data from the questionnaires was de-identified and manually transferred onto an Excel spreadsheet prior to data analysis. Any errors associated with manual data entry were verified and corrected. This was performed by cross-checking 15 randomly selected questionnaires against the original paper-based questionnaires, which accounted for more than 10 per cent of the total collected data. No errors were identified, suggesting that the risk of data entry error was low for this dataset.

### SSQ and WHODAS 2.0 scoring

Participants provided numerical responses on the SSQ questionnaire for each question, ranging from 0 (complete inability/absence of quality) to 10 (complete ability/presence of quality). Higher scores indicated lower hearing-specific disability. The scores were compared before and 12 months after CI surgery across the three SSQ domains and as a cumulative sum.

The WHODAS 2.0 questionnaire was contextualized with ‘in the last 30 days, how much difficulty did you have in…’, and participants provided numerical responses ranging from 1 (none) to 5 (extremely difficulty/cannot do). Higher WHODAS 2.0 scores indicated greater disability. The questionnaire was scored using the complex scoring method described in the official WHODAS 2.0 manual, which assigns different weight to each question. The raw data were recoded manually in Excel, and data were transferred to GraphPad Prism 8 version 8.4.2 (GraphPad Software, Boston, Massachusetts USA). The scores before and 12 months after CI were compared across each WHODAS 2.0 domain and as a cumulative sum. The life activities domain is subdivided into two subdomains: household activities and employment/work. As most participants were retired, only the subdomain related to household life activities was included in the study.

### Handling missing data

While many participants completed all questions in both questionnaires, some participants did not respond to individual questions or entire domains. As it is unclear whether partially completed questionnaires are sufficient to accurately capture each QoL domain, three approaches were employed in this study to handle missing data (Figure 1):

In approach 1, missing values were imputed for each individual question using the median response calculated from the scores of all participants for the same question.

Approach 2 followed the recommendations outlined in the official WHODAS 2.0 manual. Specifically, questionnaires missing responses for more than two different domains or missing two or more questions from the same domain were excluded from the analysis. Remaining missing values were then imputed using the median value from the remaining questions in the same domain.

In approach 3, QoL scores were adjusted to only account for questions that were answered by the participants. This approach does not introduce bias as no imputation was performed, but the entire domain may not be accurately represented as some questions are omitted.

### Statistical analysis

The Kolmogorov−Smirnov test with Lilliefors significance correction showed that the data were non-parametric. Therefore, all data were presented as the median and interquartile range (IQR) defined as the 25th to the 75th percentile. Independent Mann–Whitney U tests were performed for each relevant comparison. For matched comparisons involving only participants who completed both pre- and post-CI questionnaires, the Wilcoxon matched pairs signed rank test was performed. All tests were two tailed, and statistical significance was considered at *p*-value less than 0.05. Analysis was performed using GraphPad Prism 8 version 8.4.2 (GraphPad Software, Boston, Massachusetts USA).

### Ethics statement

This study was approved by the Western Sydney Local Health District Human Research Ethics Committee (HREC) in accordance with the National Statement of Ethical Conduct in Human Research (2007), #2019/ETH02189.

## Results and analysis

### Patient demographics

We invited 98 participants, aged 51 to 91 years, to participate in this study. The cohort had a median age of 75 years at the time of CI surgery (IQR: 67–80), and a female-to-male ratio of approximately 1:1. Amongst the 98 participants, 79 (81 per cent) completed and returned pre-CI SSQ and WHODAS 2.0 questionnaires. Only 76 of the 98 participants successfully had CI surgery. The response rate for post-CI questionnaires was slightly lower, with only 49 of the 76 (64 per cent) participants returning their SSQ questionnaires and 50 (66 per cent) returning their WHODAS 2.0 questionnaires.

### Hearing-specific QoL (SSQ) and generic QoL (WHODAS 2.0)

Using approach 1 as described in the methods, the median cumulative SSQ QoL score pre-CI was 24 (IQR: 16–35) compared to 53 (IQR: 40–64) at 12-months post-CI (*p* < 0.0001). The median cumulative WHODAS 2.0 score pre-CI was 24 (IQR: 14–39) compared to 20.5 (IQR: 10–29) 12-months post-CI (*p* = 0.053). Comparison of SSQ and WHODAS 2.0 domains pre- and post-CI are shown in Supplemental Tables 1 and 2.

**Table 1.** Pre- and 12-months post-CI QoL scores for all domains of the SSQ questionnaire, using approach 3 (non-imputation)

SSQ domain	Pre-CI score Median (IQR)	Post-CI score Median (IQR)	*p*-Value
Speech hearing	14 (7−24)	46 (26−62)	**<0.0001**
Spatial hearing	15 (7−30)	54 (28−66)	**<0.0001**
Other qualities of hearing	38 (23−52)	62 (48−69)	**<0.0001**

*Note:* Values in boldface are significant.

CI = cochlear implant; IQR = interquartile range; QoL = quality of life; SSQ = Speech, Spatial, and Qualities of Hearing Scale.

**Table 2.** Pre- and 12-months post-CI QoL scores for all domains of the WHODAS 2.0 questionnaire, using approach 3 (non-imputation)

WHODAS 2.0 domain	Pre-CI score Median (IQR)	Post-CI score Median (IQR)	*p*-Value
Cognition	25 (10−40)	15 (5−31)	**0.039**
Mobility	19 (0−44)	16 (0−49)	0.757
Self-care	0 (0−10)	0 (0−10)	0.782
Getting along	25 (8−42)	20 (6−31)	0.183
Life activities (household)	23 (0−50)	25 (0−40)	0.582
Participation in society	38 (17−54)	21 (10−38)	**0.013**

*Note:* Values in boldface are significant.

CI = cochlear implant; IQR = interquartile range; QoL = quality of life; WHODAS 2.0 = World Health Organization Disability Assessment Schedule 2.0.

Using approach 2, the median cumulative SSQ QoL score pre-CI was 24 (IQR: 16–35) compared to 52 (IQR: 36–66) at 12-months post-CI (*p* < 0.0001). The median cumulative WHODAS 2.0 score pre-CI was 23 (IQR: 12–38) compared to 17 (IQR: 6–29) 12-months post-CI (*p* = 0.041). Comparison of SSQ and WHODAS 2.0 domains pre- and post-CI are shown in Supplemental Tables 3 and 4.

**Table 3.** Subgroup analysis by gender of pre- and 12-months post-CI QoL scores for all domains of the SSQ questionnaire, using approach 3 (non-imputation)

SSQ domain	Female			Male		
	Pre-CI score Median (IQR)	Post-CI score Median (IQR)	*p*-Value	Pre-CI score Median (IQR)	Post-CI score Median (IQR)	*p*-Value
Speech hearing	16 (6−22)	44 (26−64)	**<0.0001**	16 (6−22)	44 (26−64)	**<0.0001**
Spatial hearing	14 (7−24)	52 (28−61)	**<0.0001**	14 (7−24)	52 (28−61)	**<0.0001**
Other qualities of hearing	36 (22−47)	59 (47−67)	**<0.0001**	36 (22−47)	59 (47−67)	**<0.0001**

*Note:* Values in boldface are significant.

CI = cochlear implant; IQR = interquartile range; QoL = quality of life; SSQ = Speech, Spatial, and Qualities of Hearing Scale.

**Table 4.** Subgroup analysis by age of pre- and 12-months post-CI QoL scores for all domains of the SSQ questionnaire, using approach 3 (non-imputation)

SSQ domain	< 75 Years old			≥ 75 Years old		
	Pre-CI score Median (IQR)	Post-CI score Median (IQR)	*p*-Value	Pre-CI score Median (IQR)	Post-CI score Median (IQR)	*p*-Value
Speech hearing	17 (12−23)	47 (26−63)	**<0.0001**	13 (6−27)	45 (29−58)	**<0.0001**
Spatial hearing	15 (8−24)	58 (28−67)	**<0.0001**	22 (7−32)	51 (26−65)	**0.0009**
Other qualities of hearing	33 (24−46)	58 (49−67)	**<0.0001**	43 (21−57)	63 (49−70)	**0.005**

*Note:* Values in boldface are significant.

CI = cochlear implant; IQR = interquartile range; QoL = quality of life; SSQ = Speech, Spatial, and Qualities of Hearing Scale.

Using the non-imputation approach 3, the median cumulative SSQ QoL score pre-CI was 24 (IQR: 16–35) compared to 52 (IQR: 36–66) at 12-months post-CI (*p* < 0.0001). Table 1 summarizes the pre- and post-CI median SSQ scores and IQRs for each SSQ domain. The median cumulative WHODAS 2.0 score pre-CI was 24 (IQR: 13–39) compared to 20 (IQR: 8–30) at 12 months post-CI (*p* = 0.088). Table 2 provides the scores pre- and post-CI for each WHODAS 2.0 domain.

For completeness, we also performed matched analysis using only the participants who had both pre- and post-CI questionnaires completed (Supplemental Tables 5 and 6). Significant improvement in QoL were observed in the SSQ questionnaires across all three approaches. The results for the WHODAS 2.0 were more variable; however, they collectively demonstrated that CIs do provide QoL benefits across some WHODAS 2.0 domains, including cognition and participation in society.

**Table 5.** Subgroup analysis by gender of pre- and 12-months post-CI QoL scores for all domains and cumulative score of the WHODAS 2.0 questionnaire, using approach 3 (non-imputation)

WHODAS 2.0 domain	Female			Male		
	Pre-CI score Median (IQR)	Post-CI score Median (IQR)	*p*-Value	Pre-CI score Median (IQR)	Post-CI score Median (IQR)	*p*-Value
Cognition	28 (15−44)	18 (9−37)	0.112	20 (10−40)	15 (6−29)	0.104
Mobility	25 (8−48)	38 (6−50)	0.629	7 (0−39)	13 (0−19)	0.845
Self-care	0 (0−20)	0 (0−10)	0.802	0 (0−0)	0 (0−0)	0.657
Getting along	25 (8−48)	18 (0−31)	0.155	25 (6−42)	23 (11−29)	0.608
Life activities (household)	38 (0−50)	23 (0−50)	0.393	13 (0−38)	25 (0−36)	0.837
Participation in society	42 (17−63)	30 (13−44)	0.074	35 (17−48)	21 (9−32)	**0.026**
Cumulative	31 (17−42)	24 (7−38)	0.208	22 (13−34)	15 (9−25)	0.115

*Note:* The value in boldface is significant.

CI = cochlear implant; IQR = interquartile range; QoL = quality of life; WHODAS 2.0 = World Health Organization Disability Assessment Schedule 2.0.

**Table 6.** Subgroup analysis by age of pre- and 12-months post-CI QoL scores for all domains and cumulative score of the WHODAS 2.0 questionnaire, using approach 3 (non-imputation)

WHODAS 2.0 Domain	< 75 Years old			≥ 75 Years old		
	Pre-CI score Median (IQR)	Post-CI score Median (IQR)	*p*-Value	Pre-CI score Median (IQR)	Post-CI score Median (IQR)	*p*-Value
Cognition	18(10−39)	15 (5−30)	0.235	30 (15−40)	15 (10−30)	0.104
Mobility	16 (2−47)	13 (0−50)	0.760	19 (0−41)	22 (11−44)	0.387
Self-care	0 (0−10)	0 (0−3)	0.616	0 (0−5)	0 (0−10)	0.766
Getting along	25 (0−42)	25 (8−40)	0.668	25 (8−42)	17 (8−30)	0.230
Life activities (household)	20 (0−50)	25 (0−38)	0.292	0 (0−38)	25 (0−50)	0.400
Participation in society	42 (20−55)	25 (8−41)	**0.028**	29 (13−54)	21 (11−36)	0.227
Cumulative	24 (16−38)	21 (7−30)	0.214	19 (10−37)	20 (9−27)	0.491

CI = cochlear implant; IQR = interquartile range; QoL = quality of life; WHODAS 2.0 = World Health Organization Disability Assessment Schedule 2.0.

Overall, results from all three approaches were largely similar. Since the non-imputation approach (approach 3) introduces the least bias in the context of handling incomplete data sets, we decided to use this approach for subsequent subgroup analysis.

### Hearing-specific QoL (SSQ): subgroup analysis by gender and age

Subgroup analysis by gender showed the cumulative SSQ QoL score pre-CI for females and males to be 23 (IQR: 15–31) and 25 (IQR: 19–39), respectively. Cumulative scores 12-months post-CI for females and males were 46 (IQR: 36–64) and 57 (IQR: 42–66), respectively. Both genders had significantly higher cumulative scores (*p* < 0.0001) and scores across all SSQ domains after CI (Table 3).

Subgroup analysis by age was conducted by dividing participants into two groups using the median age of 75 years as a cutoff. The median cumulative SSQ QoL score pre-CI was 23 (IQR: 19–32) for the younger than 75 years old group and 28 (IQR: 13–36) for the equal to or older than 75 years old group. The median cumulative scores post-CI were 56 (IQR: 37–65) for the younger than 75 years old group and 48 (IQR: 37–65) for the equal to or older than 75 years old group. Both age groups had significantly higher cumulative scores (*p* < 0.0001) and scores across all SSQ domains after CI (Table 4).

#### Generic QoL (WHODAS 2.0): subgroup analysis by gender and age

Subgroup analysis by gender showed no statistically significant difference between cumulative WHODAS 2.0 scores pre- and post-CI (Table 5). Males, but not females, had a statistically significant reduction in disability score for the participation in society domain (*p* = 0.026). No other statistically significant differences were observed across the domains for both genders.

Subgroup analysis by age showed no statistically significant difference between cumulative WHODAS 2.0 scores pre- and post-CI (Table 6). Participants younger than 75 years old, but not those greater than or equal to 75 years old, had a statistically significant reduction in disability score for the participation in society domain (*p* = 0.0275). No other statistically significant differences were observed across the domains for the two age groups.

## Discussion

### Interpretation of findings

This study investigated the impact of CI on QoL domains in older Australians with severe hearing loss. Hearing-specific domains, including speech hearing, spatial hearing and other qualities of hearing were significantly improved post-CI, which corroborates existing literature that also used the SSQ questionnaire.^12^ We also found significant QoL improvements in domains related to cognition and participation in society, which is not surprising as these domains are more relevant to hearing than others such as mobility.

Subgroup analysis found that hearing-specific QoL benefits derived from CI, as assessed by the SSQ, were independent of gender and age.^13^ Although objective audiometric hearing declines with increasing age, this study found that CI improved the subjective perception of hearing in both relatively older and younger adults. Although a younger participant may objectively hear better following CI, their self-reported QoL score may not improve more than an older patient, as they have different perceptions and expectations of hearing disability and its impact on daily life. These findings highlight the strength of self-reported questionnaires, compared to objective hearing performances measures, in evaluating the impact of hearing loss interventions on QoL.

Subgroup analysis of the WHODAS 2.0 questionnaire only showed QoL improvement in participation in society, and only in males and those younger than 75 years old. Hearing-impaired individuals experience numerous barriers that limit their engagement in community activities and thus could reduce their QoL, and the reasons for this disparity in QoL improvements may be better elucidated in future research.^14^^,^^15^ Another reason for this observation was that the WHODAS 2.0 questionnaire may not be sensitive to changes in one single sensory function compared to the SSQ since the WHODAS 2.0 was designed to measure disability independent of diagnosis and considers a wider range of conditions.

Our study corroborates existing literature and supports CI as an effective intervention for improving QoL in older patients with hearing loss.^3^ Age does not preclude a patient from deriving benefits from CI and, therefore, should not be a barrier. These findings could form the basis for evidence-based outcome counselling before surgery and tailored rehabilitation programs for older adults receiving CI in Australia.

### Limitations

Missing data was a limitation of this study. The self-administered nature of the questionnaire resulted in some questions being left unanswered, resulting in incomplete datasets. This could be due to participants overlooking sections of the questionnaires or their inability to understand specific questions. Future studies could administer the questionnaires via a phone or in-person interview style, to reduce the number of unanswered questions. However, this may introduce its own bias, distorting participant responses. Another cause of incomplete datasets was that a sizeable proportion of the cohort did not complete post-CI questionnaires. Potential reasons for attrition include loss of interest, participants forgetting to complete and send back questionnaires and participants passing away. Some participants did not undergo CI for various reasons and were thus ineligible to complete post-CI questionnaires.

Another limitation of the study is its relatively small cohort size. A larger sample size may allow researchers to identify minor improvements in QoL across hearing-insensitive domains. It would also allow researchers to perform more detailed analysis of the impact CIs have on QoL across different age groups and co-morbidities. Conducting larger scale studies requires significant time and financial backing, which is not always available. Our findings suggest that large-scale studies may be useful in comprehensively evaluating the impact of CI on QoL and, thus, provide a reasonable rationale for conducting large-scale, expensive studies.

Our sample size (>60 participants) was based on our power calculation where we assumed that a 30 per cent QoL benefit would be observed with CI. Using this, we found significant improvements in QoL with CI across the hearing-specific questionnaire and WHODAS 2.0 domains including ‘cognition’ and ‘participation in society’. There was a trend towards improved QoL after CI using the cumulative WHODAS 2.0 questionnaire; however, it was not statistically significant. This may be explained by the fact that the WHODAS 2.0 questionnaire assesses many domains, some of which are unrelated to hearing loss. The overall QoL improvement, as measured by the WHODAS 2.0, may be less than the 30 per cent estimate; therefore, a more conservative benefit assumption (example 20 per cent) and a larger sample size may be necessary.

Missing data required exclusion of unanswered questions in calculating questionnaire scores, described in the Methods section as approach 3. This strategy was used to minimize potential biases related to data imputation, but as unanswered questions were omitted in data analysis, this may have resulted in some domains being inadequately represented.

### Future directions

Several factors relevant to hearing loss and QoL were not investigated in the current study. These include duration of hearing loss, age of hearing loss onset, length of time CIs were used, use of hearing aids and other devices and other co-morbidities and social factors of the study participants. Future studies can provide further insight into how these factors can impact QoL following CI and whether they can be used as predictive tools for QoL outcomes in clinical practice.

This study found that the cognition domain of the WHODAS 2.0 shows significant improvement after CI. These findings concur with the studies of Sonnet *et al.*, who utilized the World Health Organization Quality of Life Questionnaire-Older Adults Module (WHOQOL-OLD) questionnaire and found that older CI recipients improved in the sensory, autonomy and executive function domains.^16^ Similarly, Calvino *et al*. reported that CI improved QoL and cognition and reduced depression scores.^17^ The Lancet Commission on Dementia Prevention, Intervention and Care report presents hearing loss as the largest known modifiable risk factor for dementia.^2^ It estimates that eliminating hearing loss could reduce dementia prevalence by 8 per cent globally. The recently published Aging and Cognitive Health Evaluation in Elders (ACHIEVE) study showed similar findings, suggesting that improved hearing may slow cognitive decline in at-risk individuals.^18^ With an aging population, research into CI as a primary prevention strategy for dementia and cognitive decline is warranted given that it may improve outcomes for many people.

## Conclusion

This study investigated the QoL benefits of CIs in older Australian adults with severe acquired hearing loss. We found evidence that CIs enhance QoL in the older populations across hearing-specific domains, as measured by the SSQ, and across domains related to cognition and participation in society. These findings may help inform future clinical practices and policies.
